# Ginger for Healthy Ageing: A Systematic Review on Current Evidence of Its Antioxidant, Anti-Inflammatory, and Anticancer Properties

**DOI:** 10.1155/2022/4748447

**Published:** 2022-05-09

**Authors:** Mehtap Ozkur, Necla Benlier, Işıl Takan, Christina Vasileiou, Alexandros G. Georgakilas, Athanasia Pavlopoulou, Zafer Cetin, Eyup Ilker Saygili

**Affiliations:** ^1^Department of Medical Pharmacology, Faculty of Medicine, SANKO University, Gaziantep, Turkey; ^2^Izmir Biomedicine and Genome Center, Balcova, Izmir 35340, Turkey; ^3^Izmir International Biomedicine and Genome Institute, Dokuz Eylül University, Balcova, Izmir 35220, Turkey; ^4^DNA Damage Laboratory, Physics Department, School of Applied Mathematical and Physical Sciences, National Technical University of Athens, 157 80 Athens, Greece; ^5^Department of Medical Biology, School of Medicine, SANKO University, Gaziantep, Turkey; ^6^Department of Biological and Biomedical Sciences, Graduate Education Institute, SANKO University, Gaziantep, Turkey; ^7^Department of Medical Biochemistry, School of Medicine, SANKO University, Gaziantep, Turkey; ^8^Department of Molecular Medicine, Graduate Education Institute, SANKO University, Gaziantep, Turkey

## Abstract

The world's population is ageing at an accelerated pace. Ageing is a natural, physiological but highly complex and multifactorial process that all species in the Tree of Life experience over time. Physical and mental disabilities, and age-related diseases, would increase along with the increasing life expectancy. Ginger (*Zingiber officinale*) is a plant that belongs to the Zingiberaceae family, native to Southeast Asia. For hundreds of years, ginger has been consumed in various ways by the natives of Asian countries, both as culinary and medicinal herb for the treatment of many diseases. Mounting evidence suggests that ginger can promote healthy ageing, reduce morbidity, and prolong healthy lifespan. Ginger, a well-known natural product, has been demonstrated to possess antioxidant, anti-inflammatory, anticancer, and antimicrobial properties, as well as an outstanding antiviral activity due to a high concentration of antiviral compounds. In this review, the current evidence on the potential role of ginger and its active compounds in the prevention of ageing is discussed.

## 1. Introduction

According to the World Population Prospects 2019, it is estimated that one in six people will be over the age of 65 (16%) by 2050 and 1 in every 11 people (9%) in 2019, respectively [1]. Ageing is a natural, physiological but highly complex, multifactorial process that all organisms go through. The life expectancy is increasing at a steady pace, and as a result, the age-related diseases as well as the physical and mental disabilities increase accordingly. While 80% of older people suffer from an age-related disease such as diabetes, hypertension, or heart disease, 50% have at least two age-related conditions. In fact, ageing has been considered as a “risk factor” for several diseases including cancer, diabetes, osteoarthritis, and cardiovascular and neurologic conditions [2, 3]. These diseases and disabilities pose a serious threat to the health of older people, thereby decreasing their quality of life and life expectancy. The biological consequences of ageing remain largely unelucidated. However, studies in the last two decades have provided valuable information on the underlying cellular and molecular mechanisms of ageing and related diseases [4, 5].

Humans have used herbs for thousands of years to treat a variety of ailments. Clinical studies and epidemiological data suggest that some plant-based natural products may reduce and delay the molecular and cellular degradation over time as much as possible and also increase longevity in humans [4]. In this context, plant-based natural products can also be promising for the prevention and treatment of ageing and age-related diseases [6–8].

## 2. Ginger

**Figure 1:**
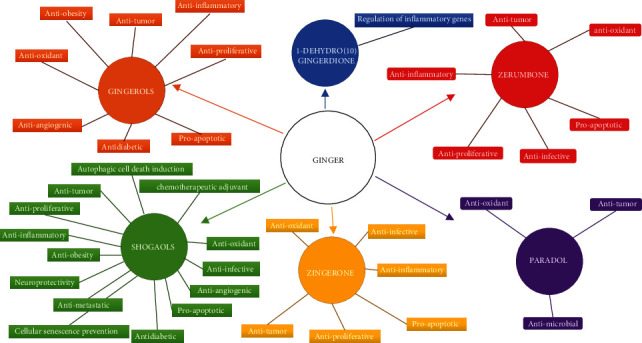
Illustration of the wide range of the health effects of ginger. A great range of those natural products' beneficial effects for the human organism have been verified and investigated at a molecular and mechanistic basis. Emphasis is given in this review on the antioxidant, anti-inflammatory, and anticarcinogenic properties of ginger.

**Figure 2:**
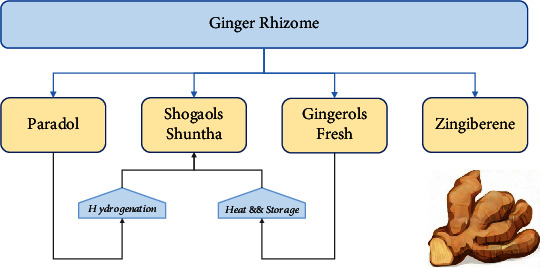
Ginger rhizome active compounds.

Ginger (*Zingiber officinale*) is a member of the Zingiberaceae plant family, native to Southeast Asia. For centuries, ginger has been consumed in different ways by Asia's indigenous peoples, mainly in China and India, both as spice and sweetener in the local cuisine and as herbal medicine for treating many diseases. Specifically, in the traditional Chinese, Indian, and Ayurvedic medicine, ginger is believed to have therapeutic effects. It is used as a remedy for cough relief due to its expectorant action to loosen and expel phlegm. Ginger is also used for pain alleviation, treatment of nausea, vomiting, and poisoning and for facilitating digestion [9]. Currently, it is known that ginger has antioxidant, anti-inflammatory, and antitumor properties and its effectiveness in the prophylaxis and treatment of gastrointestinal, cardiovascular, respiratory, and neurological diseases has been demonstrated by several research studies [10]. Rhizome is the edible part of the plant. The nutraceutical value of ginger is attributed to the bioactive compounds contained in the rhizome, such as gingerols (GNs), shogaols (SGs), paradols, and zingiberene [9]. Volatile phenolic compounds, mainly 6-GN, as well as 4-, 8-, 10-, and 12-GNs, found in fresh ginger rhizome give ginger its pungent fragrance and unique aroma. These compounds are sensitive to pH and temperature changes, and gingerols are rapidly converted to their corresponding 6-, 8-, and 10-SGs during processes that require extreme heat such as drying and roasting. Extracts derived from rhizomes and processing methods may vary widely on chemical composition and associated properties, with dried ginger as a key player in antioxidant activity. Dry ginger powder is also known as Sonth in Hindi, Sonti in Telugu, Soonth in Gujarati, Suntha in Marathi, and Shunti in Kannada. The major phenolic compounds in ginger are mainly gingerols, the active constituent of fresh ginger. The other major polyphenols are abundant in active phytochemicals such as shogaols, paradols, zerumbone, zingerone, gingerols, and 1-dehydro-(10) gingerdione. Shogaols can be derived from ginger with heat treatment or long-term storage. Paradols can form shogaols after hydrogenation. Besides these, ginger raw fiber is also involved in polysaccharides, lipids, and organic acids. Ginger active compounds are critically implicated in different biological activities such as anti-inflammatory, antitumor, antimicrobial, and antioxidant activities (Figure 1) [10]. Therefore, dried ginger rhizome represents the major source of 6-SG which is the most prominent dehydration product [11] (Figure 2). Recent studies have demonstrated that 6-SG has superior biological actions as compared to 6-GN [12, 13] with no associated side effects, and, as such, shuntha is considered more powerful medicinally than raw ginger [14, 15].

## 3. Ginger and Healthy Ageing

Active and healthy ageing is defined as the maintenance of one's ability to perform activities of daily living without being affected by cognitive and functional impairment and chronic illnesses [16]. Mounting evidence suggests that ginger can promote healthy ageing, reduce morbidity, and prolong healthy lifespan [16, 17]. For over three decades, studies have reported that the antioxidant, anti-inflammatory, antimicrobial, antitumor, and antihypercholesterolemic features of the bioactive compounds found in ginger account for its aforementioned health benefits [10, 17] (Figure 2).

**Figure 3:**
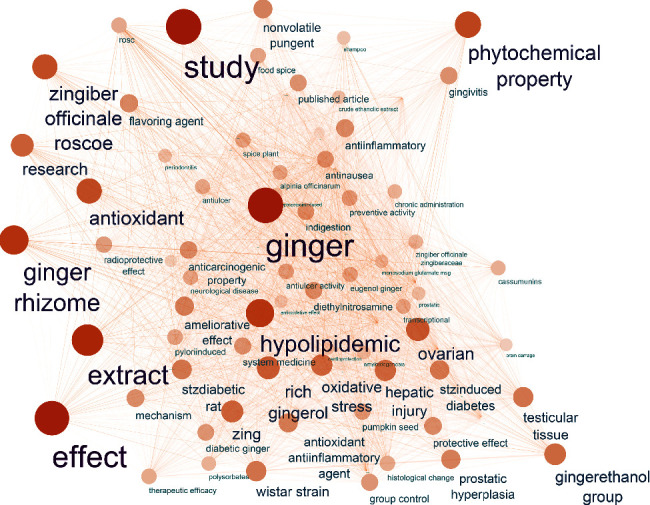
Network depicting the semantic relationships of the top 100 words most closely related to “ginger.” The words are represented by nodes, and the semantic associations between words are denoted by connecting lines.

It is believed that ginger can also protect, prevent, and even treat many diseases related to ageing by modulating molecular targets involved in their pathogenesis [6]. Furthermore, Natural Language Processing (NLP) was applied to uncover words most closely related to ginger. Briefly, PubMed literature search and article collection were conducted using the keyword “ginger”. The natural language toolkit (NLTK: https://www.nltk.org/) and spaCy (https://spacy.io/), in a Python environment, were used for text processing. The word embedding algorithm Word2vec in the Gensim library in Python (https://pypi.org/project/gensim/) was used to train word vectors in the processed text. A list of all word-to-word distances was compiled. The similarity distances between each word pair were calculated using the *Word2Vec.most_similar* function in Gensim Word2vec model. Of the 4464 candidate articles, 4009 met the inclusion criteria, such as written in English, included an abstract, and contained adequate text. Only those words that appeared more than five times (i.e., minimum frequency threshold) were vectorized. The network depicted in Figure 3 provides information on the semantic terms associated with ginger, many of them related to the health effects of ginger.

Several of the health benefits of ginger and the underlying biological mechanisms are described below.

### 3.1. Ginger against Oxidative Stress

Free radicals are highly reactive atoms or molecules with one or more unpaired electrons in their outer shells. Reactive oxygen species (ROS) are formed when oxygen reacts with certain molecules in all aerobic cells. ROS can lead to the oxidative modification of major cellular macromolecules such as carbohydrates, lipids, proteins, and DNA [18]. The free radical ageing theory, later termed as “oxidative stress theory of ageing,” is based on the hypothesis that age-related functional losses actually result from the accumulation of this oxidative damage.

Oxidative stress is associated with the reduced production of antioxidant enzymes and the activation of inflammatory pathways. Therefore, both oxidative stress and chronic inflammation lead to many molecular and cellular changes including genomic instability, epigenetic alterations, mitochondrial dysfunction, cellular senescence, and stem cell loss. These physiological/pathological changes play a key role in the development of age-related metabolic and degenerative disorders as well as cell ageing [18].

Several phytochemical extracts from ginger have been demonstrated to possess a number of antioxidant properties, like scavenging superoxide, hydroxyl, and nitric oxide radicals *in vitro* in a dose-dependent manner [19]. In one study, pretreatment with ginger extract inhibited the IL-1*β*-induced elevation of ROS and lipid peroxidation and significantly the increased gene expression of the corresponding antioxidant enzymes in C28/I2 human chondrocytes [20]. In rats, ginger showed a marked renoprotective effect on biochemical, histological, and immunohistochemical markers in nephrotoxicity induced by gentamicin and cadmium; this effect was attributed to the antioxidant and free radical scavenging activities of polyphenolic compounds such as 6-GN and 6-SG [21, 22]. Exogenous 6-SG and 6-, 8-, and 10-GN exhibited considerable antioxidant activities, with IC_50_ values ranging from 8.05 to 26.3 *μ*M against DPPH (1,1-diphenyl-2-picyrlhydrazyl) radical, 0.85-4.05 *μ*M against superoxide radicals, and 0.72-4.62 *μ*M against hydroxyl radicals. In that study, 6-SG was reported as the most potent antioxidant and anti-inflammatory compound, with 6-GN exhibiting the least antioxidant activity [23].

In addition to antioxidant activity, the bioactive components of ginger can also exert a modulating effect on antioxidant enzymes or enzymatic systems. In a study investigating the effect of ginger extract on oxidative hepatic toxicity induced by lead acetate (PbAc) in rats, the extract reduced the levels of malondialdehyde (MDA), a marker of oxidative stress in the liver, and caused a significant increase both in the concentration (by 112-228%) and mRNA expression (1.4- to 8-fold) of antioxidant enzymes and molecules including hepatic glutathione, glutathione S-transferase (GST), glutathione peroxidase (GPx), and catalase (CAT) [24]. Ginger extract was reported to decrease the MDA level in the liver and also restore the activity of antioxidant enzymes including GPx, glutathione reductase (GR), superoxide dismutase (SOD), and CAT and the hepatic content of reduced glutathione when orally administered to rats with streptozotocin-induced diabetes mellitus for 30 days [25]. In a study examining the effectiveness of 6-GN on the longevity of *Caenorhabditis elegans*, SOD and CAT activities were increased by 21.8% and 28.3%, respectively, in worms fed 6-GN versus controls, whereas intracellular ROS levels decreased in a dose-dependent manner [26].

The antioxidant content and composition of plant extracts vary depending on a number of factors including extraction solvent, temperature and duration of extraction, and storage conditions [27]. Higher antioxidant activity was observed in ginger extracts prepared with ethanol, methanol, and acetone solvents than extracts prepared with water [28, 29]. In a study using a number of free radical scavenging methods, ginger root showed the greatest scavenging activity against hydroxyl (HO^·^) and superoxide radicals. Following storage of the plant at 80°C for two hours, its effect on singlet oxygen (^1^O_2_) and peroxyl radical decreased by 50%, whereas its effect on superoxide radical increased by 56%. This heat-induced change in the radical scavenging activity was attributed to the conversion of 6-GN, the main ginger component, to 6-SG [30]. This piece of evidence indicates that heat treatment may either increase or diminish the scavenging activity of ginger depending on the type of reactive oxygen species. This suggests that, unlike other vegetables with antioxidants, ginger will not lose its antioxidant activity even after cooking at high temperatures [30].

Although the drying process results in degradation of some of the cellular components of the ginger rhizome, it also induces the formation of new compounds with enhanced antioxidant properties. In particular, the antioxidant properties of the dried ginger tea were found to be superior to those of the commercial tea [31]. Of importance, the drinking of ginger tea can effectively prevent and cure colds, sore throat, and cough and also reduce inflammation [32]. In a study exploring the effects of different drying methods including sun drying (at 28-44°C for 3 days), oven drying (at 60°C for 4 days), and freeze drying (at -30°C for 3 days) on the antioxidant and anti-inflammatory activities of ginger rhizome, all drying processes, primarily sun drying, were reported to enhance the aforementioned properties of the plant significantly as compared to fresh ginger [33].

In a study examining the effects of storage time and temperature on the activities of ginger phytochemical compounds such as phenolics, flavonoids, 6-GN, and 6-SG, storage temperature was observed to affect free radical scavenging activity to a greater extent than storage time. It has been suggested that when stored at 5°C, the qualities of fresh rhizome can be preserved for up to 4 months in terms of both antioxidant and antibacterial activities [34].

There is a growing interest globally in organic farming because organic foods are considered healthier. In a study by Min and colleagues, it was found that organic ginger rhizomes and leaves have a significantly greater phytochemical and antioxidant capacity than their nonorganic counterparts. Moreover, applications of mycorrhiza combined with tissue culture for organic farming resulted in a marked increase in the total phenolic and flavonoid contents, as well as 6-GN and 8-gingerol concentrations, in ginger rhizomes [35].

### 3.2. Anti-Inflammatory and Immunomodulatory Activities

Inflammation can be defined as the body's protective response that develops following invasion by microorganisms, antigen exposure, and damage to cells and tissues. It involves complex interactions among many cell types, mediators, receptors, and signaling pathways. In particular, chronic inflammation has a central role in the pathogenesis of many diseases such as atherosclerosis, cancer, diabetes, and rheumatoid arthritis as well as ageing. Studies have long shown that ginger and its various active compounds have anti-inflammatory activity. It was initially suggested that the anti-inflammatory activity of ginger is mainly associated with its inhibitory action on prostaglandin and leukotriene synthesis [15]. Both fresh ginger (mainly composed of gingerols) and dried ginger extracts (major source of shogaols) were demonstrated to inhibit lipopolysaccharide- (LPS-) induced prostaglandin E2 (PGE2) production [36, 37]. This activity of 6-, 8-, and 10- gingerols was attributed to their inhibitory action on *COX-2* mRNA expression and the corresponding COX-2 enzyme, with 10-gingerol exhibiting the strongest inhibitory effect depending on the length of the side chains. However, 6-, 9-, and 10-shogaols were found to have lesser inhibitory effect on *COX-2* mRNA expression [38].

In a carrageenan-induced rat paw edema model in which the anti-inflammatory activity of ginger extract was evaluated at different doses, the extract was shown to reduce the levels of the inflammatory mediators PGE2, TNF-*α*, IL-6, monocyte chemoattractant protein-1 (MCP-1), and myeloperoxidase (MPO) by 32% to 60% in a dose-dependent manner (25-200 mg/kg). The anti-inflammatory activity of ginger extract was found to be significantly greater than that of diclofenac at the same concentration, and 6-SG was more potent than other shogaols. At a dose of 200 mg/kg, ginger extract prevented histopathological effects induced by carrageenan and increased total antioxidant capacity. The authors suggested that the anti-inflammatory activity of ginger extract mainly involves inhibition of cell migration and activation of inflammatory cells [39].

Several studies showed that 6-GN (i) inhibited inducible nitric oxide synthase (iNOS) and TNF-*α* expression in mouse macrophages stimulated by LPS, (ii) reduced IL-6, IL-8, and ROS levels in hepatic HuH7 cells stimulated by IL-1*β*, (iii) decreased IL-8, IL-6, IL-1*β* protein, and mRNA levels in intestinal epithelial cell inflammation induced by *Vibrio cholerae*, (iv) reduced secretion and expression of TNF-*α*, IL-1*β*, IL-6, PGE2, COX-2, and iNOS in murine RAW 264.7 monocyte/macrophage-like cells exposed to LPS, and (v) downregulated expression of iNOS and COX-2 protein in murine skin cells exposed to 12-O-tetradecanoylphorbol 13-acetate (TPA) by either modulating or inhibiting a NF-ĸB- (nuclear factor kappa-light-chain-enhancer of activated B cells-) mediated signaling pathway or via its interaction with various endogenous mediators such as PI3K/Akt/I kappaB kinases (IKK) and MAPK [40–44]. In addition, the inhibitory effect of 6-GN on COX-2 enzyme has been reported to contribute to its anti-inflammatory activity [41].

Systemic administration of 6-GN caused hypothermia by slowing the metabolic rate in rats and led to inhibition of acetic acid-induced writhing response and formalin-induced licking time in mice. These data suggest that 6-GN may potentially have antipyretic and analgesic properties as well as anti-inflammatory activity [45, 46].

It was reported that 6-SG inhibited the release of TNF-*α*, IL-1*β*, and NO in LPS-activated RAW264.7 macrophages, suggesting that it may have a therapeutic potential in chronic inflammatory events primarily involving macrophages [47]. It was also shown to inhibit activation of a NF-ĸB-mediated pathway and subsequent release of TNF-*α*, IL-1*β*, IL-6, and PGE2 in BV2 microglia cells exposed to LPS. Upregulation of peroxisome proliferator-activated receptor gamma (PPAR*-γ*) by 6-SG was found to play a role in its anti-inflammatory action [48].

Toll-like receptors (TLRs) play a crucial role in innate immunity. TLRs are expressed by the elements of the innate body defense system such as macrophages and dendritic cells and trigger the immune system following breaching of physical barriers, such as skin or intestinal mucosa, by microorganisms. During this process, myeloid differentiation factor 88 (MyD88) and Toll-interleukin-1 (IL-1) receptor domain-containing adapter inducing interferon beta (TRIF) act as signal transducers. In one study by Park et al., 6-SG was shown to inhibit activation of NF-ĸB and IRF3, as well as their target genes, *COX2* and *IFN-β*, by suppressing the TRIF pathway of TLR3 and TLR4 [49]. These data provide important clues for elucidating the anti-inflammatory mechanism(s) of action of 6-SG in bacterial and viral infections. Moreover, the antipyretic and analgesic effects of 6-GN, as compared to 6-SG, were found to be more potent, when both were administered intravenously at doses ranging from 1.75 to 3.5 mg/kg and orally at doses from 70 to 140 mg/kg [50].

According to two recently published meta-analyses, where the effects of ginger supplementation on inflammatory markers in humans were examined, significantly lower levels of serum CRP, TNF-alpha, IL-6, and PGE2 were reported within a period of 2-3 months versus controls [51, 52].

In male endurance runners, intake of 500 mg capsules of ginger powder during a 6-week vigorous training period significantly lowered the postexercise levels of the proinflammatory cytokines IL-1*β*, IL-6, and TNF-*α*, known as “indicators of athletes' acute immune function,” and also reduced the fatigue-like symptoms induced by these cytokines [53].

### 3.3. DNA Damage and Ginger's Anticancer Activity

Data in literature are mainly focused on the actions of a ginger extract, the polyphenolic alkenone, 6-GN [2, 54]. Anticancer, anti-inflammatory, antitumor, and proapoptotic activities have been identified as potential effects of 6-GN. Relevant effects have been found in a variety of cancers [55–62]. The underlying biological pathways include, but are not limited to, DNA damage-associated cell cycle regulation, apoptosis, and chromatin regulation. These effects, in combination with the absence of toxicity in healthy cells, can have promising future use in clinical setup underlining its differential effect on cancer and normal cells.

Results from human cervical cancer cells *in vitro* and *in vivo* have shown that 6-GN treatment may significantly increase DNA damage [63]. Results from the sensitive and reliable biomarker of gamma-H2AX have shown that the main pathway depends on the generation of intracellular ROS, which led to p53 reactivation followed by the p53-mediated G2/M cell cycle arrest. Amplification of apoptotic effects of cisplatin also occurred, presumably due to ROS-mediated induction of DNA damage and increased apoptotic effects. Similar results are also present in both acute and chronic myeloid leukemia cell lines, as examined via p-H2AX and comet assay in Rastogi et al.'s study [54]. Increased levels of the phosphorylated histone do confirm significant underlying DNA damage. Cell viability assays were also performed in this study where ROS-mediated DNA damage resulted in G2/M cell cycle arrest. This was further validated with flow cytometry-based TUNEL assay. Significant cell cycle arrest at G2/M checkpoint has also been observed in human colon cancer cell lines; the increased population of cells in G2 phase in combination with p53 phosphorylation indicates increased DNA damage effects [64]. Apart from 6-GN, another phenolic agent extract from ginger, zingerone, has shown comparable effects in colon cancer cells. Comet assay results indicate a significant increase in DNA damage following zingerone treatment in these cell lines, leading to decreased cell proliferation and proapoptotic effects. Herein, there is a possible speculation on the involvement of the ROS-mediated apoptosis pathway.

A different experimental setup has been employed in murine sarcoma cell lines by Lima et al. [57], where decreased cell viability was observed in high concentration of 6-GN compared to another antineoplastic agent. In this study, a cytogenetic approach of micronuclei formation was employed, where at high concentrations nucleoplasmic bridges and nuclear buds were observed with concomitant increase in apoptosis. Interestingly, pretreatment with 6-GN may also enhance IR-induced cell apoptosis in gastric cancer cell lines [55]. This piece of evidence, in combination with cell proliferation inhibition and increased cell radio sensitization, can be taken into consideration in the combined therapeutic effects of chemoradiotherapy. Radiosensitization in cancer cell lines has also been reported in colorectal cancer cell lines, where Zerumbone pretreatment is associated with radiation-induced cell cycle arrest and apoptosis [65]. Concurrently, the dose and route of administration-dependent (comparing to earlier studies) radioprotective effects in mice have been reported for another hydroalcoholic extract of ginger rhizome, zinger. Radiation toxicity syndromes and associated lethality presented ameliorated effects in whole body gamma irradiation [66]. 10-Gingerol (10-GN) reports, albeit limited, are aiming towards similar direction as 6-GN. Decreased cell proliferation and accumulation of cells in S phase (cell cycle arrest) were reported in TNBC [67] and human colon cancer cells [68].

On the other hand, the results on the influence of simple dietary change, where mice were pretreated with samples of a lyophilized extract of rhizome ginger (i.e., hydroalcoholic extraction containing ~2.54% gingerols), are not in agreement with previous reports on the inhibition of bladder cancer induced by N-butyl-N-(4-hydroxibutyl) nitrosamine mouse bladder tumors [67].

Studies on ginger spice as supplement (i.e., powder ethanol—dried) have demonstrated the protective effects and decrease in DNA damage induced by hydrogen peroxide in 3T3-L1 fibroblasts. Ginger, as compared to other dietary supplements, showed significant DNA protective activity in various concentrations and also the overall maximum antioxidant activity [69].

The incidence rate of the majority of cancers is increasing with age (https://www.cancer.gov/about-cancer/causes-prevention/risk/age). Despite extensive research efforts on elucidating the molecular basis of cancer and developing strategies for its accurate diagnosis and effective treatment, cancer remains a major health problem with a rapidly increasing incidence. This has attracted interest for functional foods that are believed to have cancer preventive and tumor growth retarding potential. Over the last 25 years, many studies have demonstrated chemopreventive potential for the active compounds of ginger such as 6-GN and 6-SG in animal models, cell lines, and human cancers. The antineoplastic activities of these compounds have been associated with their antioxidant, anti-inflammatory, antiangiogenic, antiproliferative, and proapoptotic features.

Similarly, an active ingredient of ginger, 6-SG, has been found to inhibit breast cancer cell invasion, by reducing matrix metalloproteinase-9 expression via blockade of NF-*κ*B activation. In addition, 6-SG was demonstrated to induce apoptosis in hepatocellular carcinoma, NB4, MOLT4, and Raji leukemia cells and prostate cancer cells by modulating endoplasmic reticulum stress and a caspase-3-mediated pathway, p53, BAX and BCL2, and STAT3 pathways, respectively [70–72]. Recently, Bawadood et al. suggested that 6-SG suppressed autophagy in breast cancer cells and also induced apoptosis by targeting the NOTCH signaling pathway [73].

However, a recent study demonstrated that ginger extract suppresses progression of cell cycle in the human pancreatic PANC-1 cancer cell line and induces autotic cell death. Unlike apoptosis and necroptosis, autosis results in a variety of morphological changes in cancer cells including nuclear shrinking, focal membrane rupture, electron-dense mitochondria, and vacuolation. In the same study, both the ginger extract and 6-SG were reported to increase the LC3-II/LC3-I ratio and decrease SQSTM1/p62 expression and, at the same time, activate AMPK and inhibit mTOR, all of which are indicators of autophagy. The authors concluded that ginger exerts its anticancer activity by inducing the formation of ROS in cancer cells [74]. Similarly, 6-SG was shown to induce autophagic cell death by promoting LC3 expression in breast cancer cells and by inhibiting the AKT/mTOR pathway in non-small-cell lung cancer A549 cells [75, 76].

Although the findings of these studies seem to contradict those of previous studies, data suggest that autophagy is a strategic survival mechanism, that is, cancer cells may have turned to autophagy to escape apoptotic death [75].

It was considered that PTHrP, which is normally secreted by some cancer cells, may promote metastasis to the bone by interacting with osteoblasts and osteoclasts [77]. The chemical agent 2-amino-1-methyl-6-phenylimidazo [4,5-b] pyridine (PhIP) is found in processed and red meat and is regarded as a potential carcinogen for renal cell carcinoma (RCC). Human 786-O renal cell carcinoma cells upon induction by PhIP showed increased secretion of both parathyroid hormone-related protein (PTHrP) and IL-8. Subsequently, cultures of human osteoblasts with PhIP-stimulated condition medium of 786-O increased the expression of the macrophage colony-stimulating factor (M-CSF) and receptor activator of NF-ĸB ligand (RANKL) and decreased the expression of osteoprotegerin (OPG). It was reported that 6-SG decreased PTHrP and IL-8 expression in RCC cells and decreased RANK expression in osteoblasts. Overall, these findings demonstrate that PhIP is an important risk factor for bone metastasis in RCC and may enhance osteoclastic bone resorption, suggesting that 6-SG could exhibit antimetastatic activity by blocking PhIP-induced bone resorption in an *in vitro* RCC model [77].

Meanwhile, it was observed that 6-GN, the other bioactive compound of ginger, downregulates the *NF-ĸB*, *AKT*, and *Bcl2* genes in HeLa cells and increases the expression of *TNFα*, *BAX*, and *cytochrome c* as well as *caspase-3* and *PARP*. These data suggest that 6-GN induces death of cancer cells possibly through caspase-3-mediated apoptosis and autophagy [78]. In another study, treatment with 6-GN inhibited cell growth and colony formation in a time- and dose-dependent manner in renal cell carcinoma, by exerting its effect on the AKT-GSK 3*β*-cyclin D1 signaling pathway, and caused cell cycle arrest at the G1 phase [79].

Benzo(a)pyrene intake is known to play a pivotal role in the development of colorectal cancer in humans. In a murine colorectal cancer model induced by benzo(a)pyrene, 6-GN was shown to inhibit tumorigenesis via its anti-inflammatory, antiproliferative, antiangiogenic, and apoptotic actions [80]. Moreover, 6-GN was reported to exhibit anti-inflammatory activity by decreasing *TNF-α*, *Il-1β*, *INOS*, and *COX-2* expression and also inhibit cell proliferation by increasing the expression of *WNT3a*, *DVL-2*, and *β-catenin* and, conversely, decreasing *cyclin D1* and *Ki 67* expression. In the same study, 6-GN displayed antiangiogenic activity by reducing the expression of *VEGF*, *angiopoietin-1*, *FGF*, and *GDF-15* and also apoptotic activity by increasing *APC* and *p53* expression [80]. A semisynthetic derivative of 6-GN (i.e., SSi6) was reported to both inhibit migration and invasion of cancer cells and exhibit cytotoxic effect on human epithelial-like breast cancer cells [81].

#### 3.3.1. Ginger Supplements

In a population of humans at high risk for colorectal cancer, supplementation with ginger extract at a dose of 2 g/day for 28 days reduced the expression of the telomerase reverse transcriptase (hTERT) and MIB-1 (epitope of Ki-67), two markers of cell proliferation, and elevated the expression of the *BAX* gene (an indicator of apoptosis) as demonstrated by biopsy samples of colon mucosa [82]. In another study, supplementation with ginger extract was found to decrease *COX-1* expression in individuals at increased risk for developing colorectal cancer. It is known that the COX-1 enzyme has an integral role in the synthesis of PGE2, which is primarily involved in the development of colorectal cancer. This finding suggests that ginger supplementation may lower the risk for developing colorectal cancer through its COX-1 inhibitory effect [83].

#### 3.3.2. Ginger in Antineoplastic Combination Therapies

Combination therapy with two or more drugs or agents can elicit a synergistic and/or additive effect in the treatment of cancer and reduce drug resistance and drug toxicity. The combination of *γ*-tocotrienol and 6-GN was shown to induce cytotoxicity and apoptosis synergistically in HT-29 and SW837 human colorectal cancer cells [84]. In another study conducted by the same research team, the combination of ginger extract and Gelam honey was reported to exhibit chemopreventive activity in HT29 colon cancer cells by modulating the Ras/ERK and PI3K/AKT pathways in a synergistic manner [85]. Additionally, treatment with a combination of 6-GN and epigallocatechin gallate (EGCG), the latter with reported antineoplastic properties, was shown to synergistically induce apoptosis and inhibit growth of cancer cells [86].

In a murine breast cancer model, Ashmawy et al. demonstrated that combined use of ginger extract with doxorubicin (DOX) increases the survival rate of mice as compared to the group receiving DOX alone, reduces tumor mass, and increases apoptosis [87]. Recently, 6-SG was shown to increase the antineoplastic efficacy of the anticancer drugs 5-fluorouracil, oxaliplatin, and irinotecan in SW480 and SW620 colon cancer cell lines by increasing their ability to induce apoptosis and autophagy [88]. The aforementioned data suggest that addition of ginger extract or 6-SG in conventional chemotherapeutic regimens may improve treatment outcomes [87, 88].

#### 3.3.3. Ginger in Nausea and Vomiting Induced by Antineoplastic Drugs

Nausea and vomiting are the most frequent and bothersome side effects experienced by cancer patients receiving chemotherapy. Despite the availability of effective drug and antineoplastic combination therapies in recent years, the treatment success rate is not adequate [89]. Several animal and human studies reported that ginger extract can be effective in the management of chemotherapy-induced nausea and vomiting (CINV) [90]. CINV is classified as both acute and delayed side effect according to the time of onset. In two recent meta-analyses, ginger (in the form of oral extracts or capsules) was found to be more effective in preventing acute nausea and vomiting in patients receiving chemotherapy treatment [91]. It was concluded that its antiemetic effect was more pronounced especially when ginger supplementation was received at a maximum dose of 1 g for more than 3 days, starting from the first day of chemotherapy [92]. The mechanism of action of ginger and its active components in CINV remain unclear. Studies have reported that ginger shows an antagonistic action by binding to a distinct site on 5HT-3 receptors, other than the sites that antiemetic drugs (e.g., ondansetron) bind to and, therefore, may elicit a synergistic inhibitory effect when administered with 5HT-3 receptor antagonists [90]. Additionally, many preclinical studies demonstrated that the inhibitor activity of ginger in CINV is mediated by its antagonistic effects on neurokinin-1 and dopamine receptors, together with its antioxidant and anti-inflammatory actions [90].

### 3.4. Neuroprotective Activity

The prevalence of neurodegenerative diseases such as Alzheimer's disease, Parkinson's disease, and dementia increases with ageing. Recent studies have suggested that ginger may have neuroprotective effects on these chronic, noncurable disorders mediated by mechanisms other than those underlying its known antioxidant, anti-inflammatory, and antiapoptotic properties [6].

#### 3.4.1. Alzheimer's Disease

Alzheimer's disease (AD) is the most common neurodegenerative disorder in the elderly. AD is a progressive disorder and one of the major causes of dementia in this age group. It is characterized by *β*-amyloid protein deposition leading to plaque formation, aggregates of hyperphosphorylated *tau* protein that form tangles of neurons, oxidative stress, and reduction in acetylcholinesterase (AChE) levels in certain areas of the brain [4].

In a rat model of AD *induced by* oral aluminum chloride and injection of intracerebroventricular *β*-amyloid protein, ginger extract was shown to increase SOD and CAT expression in the brain and decrease secretion and expression of NF-ĸB, IL-1*β*, and MDA levels, leading in this way to improvement of the behavioral dysfunction [93]. In a murine model of AD induced by injection of *β-*amyloid plaques, fermented ginger (to increase bioavailability) was reported to significantly reduce synaptic disorder and neuron cell loss as compared to nonfermented ginger [94]. In a separate study, ginger extract was found to inhibit AChE activity as well as lipid peroxidation induced by administration of prooxidant substances in the rat brain [95].

Cholinergic neuron loss in the hippocampus is associated with memory loss and attention problems in AD. In an HT22 hippocampal neuron cell culture known to express cholinergic markers, 6-SG reduced the production of hydrogen peroxide (H_2_O_2_) ROS and increased cholinergic activity. The brain-derived neurotrophic factor has been suggested to contribute to the neuroprotective effect of 6-SG [96]. In a mouse model of AD induced by *β-*amyloid protein, 6-SG was shown to exert neuroprotective effects via its antagonistic action against the cysteinyl leukotriene 1 receptor, which is known to have a central role in the pathogenesis of AD [94]. In mouse hippocampal HT22 cells, 6-SG activated sortilin-related receptor 1 (SORL1), which is reported to decrease amyloid precursor protein in the brain [97].

Additionally, by investigating the effects of 6-GN on cytotoxicity and apoptotic cell death induced by *β-*amyloid in rat pheochromocytoma cells (PC12 cells) and human neuroblastoma SH-SY5Y cells, 6-GN was observed to decrease ROS levels and increase the antioxidant enzyme activity in these cells [98, 99]. In middle-aged women, ginger extract supplementation was found to improve cognitive function when received at a dose of 400-800 mg daily [100].

#### 3.4.2. Parkinson's Disease

Parkinson's disease (PD) is a neurodegenerative disorder characterized by a progressive loss of dopamine-producing neurons in certain regions of the brain with ageing [6].

In a murine model of PD induced by 1-methyl-4-phenyl-4-propionoxypiperidine (MPTP), ginger extract was shown to protect neurons against apoptosis, increase dopamine levels in the globus pallidus and striatum, and reduce TNF-*α*, NO, and ROS levels, resulting in this way in improved PD symptoms of motor coordination disorder and bradykinesia [101]. In another study, 6-SG was reported to exert neuroprotective effects *in vivo* (C57/BL cells) and *in vitro* (rat mesencephalic cell cultures) PD models [102].

Recent studies have demonstrated that the treatment of intestinal dysfunction is important in neurodegenerative disorders such as PD. In C57BL/6J mice (i.e., resistant to audiogenic seizures) with intestinal damage induced by MPTP, ginger and 6-SG suppressed the elevation of NOS, TNF-*α*, and Il-1*β*, exhibited protective effects on enteric dopaminergic neurons, and maintained intestinal integrity [103].

### 3.5. Cardioprotective Activity

Ageing is one of the leading risk factors for developing cardiovascular diseases. It is known that dyslipidemia and hypertension are important predisposing factors for many cardiovascular conditions including coronary heart disease and stroke. Recent studies have shown that ginger and some of its active constituents may be useful in lowering blood lipid levels and blood pressure and preventing platelet aggregation.

It was reported that intravenous administration of fresh ginger extract lowers blood pressure in anesthetized rats, and this activity was attributed to its inhibitory effect on voltage-dependent calcium channels [104]. In a rat model of hypertension induced by the NOS inhibitor L-NAME, pretreatment with ginger rhizomes was shown to lower blood pressure, inhibit the angiotensin-1-converting enzyme (ACE) and arginase activities, and increase vasodilator nitric oxide (NO) levels [105]. Another study showed that 6-GN inhibits the activation of angiotensin II type 1 receptors [106]. As it is known, ACE converts angiotensin I to angiotensin II, which is a potent vasoconstrictor peptide primarily involved in the pathogenesis of hypertension and exerts its effect via angiotensin II type 1 receptors. In a study where several *in vitro* cell cultures were used, 6-GN was reported to normalize the expression of major biomarkers related to hypertension through the peroxisome proliferator-activated receptor delta (PPAR*δ*) [107]. Recently, in a rat hypertension model induced by L-NAME, *Zingiber officinale* was reported to increase both the antihypertensive effectiveness and plasma concentration of losartan, an antihypertensive drug and an angiotensin II type 1 receptor antagonist [108]. These data suggest that ginger may also act on hepatic microsomal enzymes that are involved in the metabolism of some antihypertensive drugs.

In a meta-analysis of clinical trials, it was concluded that ginger supplementation at a dose of >3 g daily for two months is effective in lowering blood pressure in middle-aged individuals [109].

Elevation of blood lipids is considered the leading cause of atherosclerosis. In rats fed a high-fat diet, the combination of aerobic exercise and ginger extract resulted in a significant reduction in serum triglyceride (TG), low-density lipoprotein (LDL), and total cholesterol levels and a marked increase in the levels of high-density lipoprotein (HDL); these observations suggest that ginger may confer protection against atherosclerosis [110]. Of note, in recent years, it has been reported that oxidation of apoA-I, a component of HDL, may result in the production of dysfunctional HDL in hyperlipidemic states. In one study, ginger extract was found to stimulate functional HDL production by restoring apoA-I function via inhibition of oxidative stress in hamsters fed a high-fat diet. In the same study, it was also found that ginger increased fecal excretion of cholesterol [111].

Transforming-growth factor beta (TGF-*β*) increases the binding potential of LDL, by inducing proteoglycan synthesis in vascular smooth muscle cells. Because of this activity, which has a crucial role in the development of atherosclerosis, TGF-*β* exhibits also proatherogenic properties. In one study, it was suggested that 6-GN may have a protective role against the development of atherosclerosis by inhibiting proteoglycan synthesis [112].

Many recent studies have demonstrated blood-lowering effects of ginger supplementation in humans. A significant reduction in serum/cholesterol levels was reported in hyperlipidemic patients who were given ginger supplement at a dose of 3 g/day for 45 days in one study and for 4 weeks in another study [113, 114].

Platelet aggregation is a well-established risk factor for coronary heart disease and stroke. Recently, potent *in vitro* antiplatelet activity of ginger was demonstrated in platelet aggregation stimulated by adenosine 5-diphosphate (ADP), bovine thrombin, and arachidonic acid as compared to aspirin (positive control) [115].

### 3.6. Endocrine Diseases

Age is the major risk factor for the development of type 2 diabetes mellitus (DM). The pathogenesis of DM involves insulin resistance and pancreatic beta cell dysfunction.

In type 2 diabetic Lepr^db/db^ mice, 6-GN was shown to increase glucose-stimulated insulin secretion, which was mediated by glucagon-like peptide-1 (GLP-1). In the same study, 6-GN was reported to increase the presence of glucose transporter type 4 (GLUT-4) molecules in skeletal muscle cells and facilitate glycogen storage, resulting in this way to improvement of hyperglycemia [116].

In diabetes, long-standing hyperglycemia is known to induce protein glycation, which is associated with covalent binding of glucose to plasma proteins. In turn, protein glycation results in the formation of advanced glycation end products (AGEs), which accelerate the development of diabetic complications such as nephropathy, retinopathy, neuropathy, and cardiomyopathy. In an *in vitro* study, 6-GN and 6-SG were shown to inhibit protein glycation by trapping methylglyoxal, a highly reactive AGE precursor in diabetes [117].

Vascular calcification plays an important role in the morbidity and mortality in diabetic patients, due to associated cardiovascular complications. Recently, 6-SG has been shown to antagonize hyperglycemia-induced vascular calcification by inhibiting the Akt/ROS signaling pathway and NLRP3/caspase 1/IL-1*β* inflammasome in human arterial smooth muscle cells [118]. In addition, treatment with ginger extract and 6-SG was reported to alleviate pain associated with diabetic neuropathy via its effects at the spinal cord [119].

In a recent meta-analysis, regular ginger supplementation (3 grams daily in some studies) [6] was reported to reduce fasting blood glucose concentration, insulin resistance, and HbA1c level in type 2 DM patients [120].

Collectively, these data suggest that ginger and its active components may have beneficial effects on the short- and long-term control of blood glucose, insulin resistance, and protective effects against the development of vascular complications in diabetes.

Obesity is an important risk factor for diabetes, hypertension, and many cardiovascular diseases, as well as an important metabolic disease that accelerates the ageing process.

PPAR*δ* is the main regulator of energy metabolism in skeletal muscle and adipose tissue. Activation of PPAR*δ* has been shown to induce fatty acid oxidation and reduce obesity in mice [121]. In one study, ginger extract reduced diet-induced obesity in mice and increased exercise endurance capacity by increasing skeletal muscle fat catabolism; it was suggested that this effect may be mediated by the modulatory actions of 6-SG and 6-GN on the PPAR*δ* signaling pathway [122].

Recent studies suggest that gut microbiota may represent an important target in the treatment of obesity. In a recent study, ginger supplementation has been reported to reduce body weight, fatty liver, and insulin resistance by restoring gut microbiota in rats fed a high-fat diet [123].

Steamed ginger ethanolic extract (SGE) is rich in 6-SG. A marked reduction in average body weight, body mass index, and body fat content was observed in healthy obese individuals taking 100 mg SGE supplementation daily for 12 weeks [107, 124]. These findings suggest that supplementation with a ginger extract rich in 6-SG along with lifestyle changes, such as diet control and/or physical activity, may have a significant potential in lowering body mass index [124].

### 3.7. Anti-Infective Actions

Reduced immune function and comorbidities related to ageing increase the risk for developing infections in older people. Recent studies have shown that ginger and its bioactive constituents have antibacterial, antifungal, and antiviral activities. In particular, ginger and/or its active components were reported to be active against drug-resistant bacteria such as *Escherichia coli*, *Salmonella typhi*, *Staphylococcus aureus*, *Pseudomonas aeruginosa*, *Mycobacterium tuberculosis*, and *Enterococcus faecalis* and fungi such as *Candida albicans* [14, 125, 126].

The cytokine interferon-*γ* (IFN-*γ*), which is produced by natural killer cells and activated by T lymphocytes, has a critical role in protecting our body against viral and bacterial infections. 6-SG was found to enhance IFN-*γ* transcription and expression in a dose-dependent manner in human T lymphocyte cells [127].

### 3.8. Ginger and COVID-19

Ginger has been demonstrated to have an outstanding antiviral activity due to a high concentration of antiviral compounds [14, 128]. Coronavirus disease 2019 (COVID-19) is an infectious respiratory tract disease caused by the severe acute respiratory syndrome coronavirus 2 (SARS-CoV-2). Since its discovery in 2019, the virus has spread worldwide, causing the COVID-19 pandemic. The disease can be fatal especially in elderly infected patients with comorbidities [129]. Although there is no definitive treatment or a fully effective vaccine for COVID-19, extensive research is ongoing. The viral nonstructural protein 15 (Nsp15) is increasingly being considered as an important therapeutic target for the viral replication of SARS-CoV-2. In a recent *in vitro* study using hydroxychloroquine (one of the few drugs recommended for the management of COVID-19) as a positive control, it was suggested that gingerol can inhibit viral replication by binding to the Nsp15 viral protein of SARS-CoV-2 [130]. Additionally, in a very recent publication, it was stated that ginger extract may be used as an adjunctive treatment for COVID-19 due to its demonstrated beneficial effects in acute respiratory distress syndrome (ARDS), pulmonary fibrosis, pneumonia, and sepsis, which also occur in COVID-19 patients [131]. In 2020, Ahkam et al. designed an in silico study to investigate the potential utilization of the antiviral properties of ginger in counteracting SARS-CoV-2 infection based on the interaction of ligand ginger compounds with the viral spike (S) protein and main protease (MPro) [132]. Thus, a promising therapeutic strategy would be to develop structure-dependent antiviral drugs based on phytochemical compounds that inhibit essential SARS-CoV-2 proteins.

### 3.9. Sarcopenia

Sarcopenia is an age-related condition characterized by reduced skeletal muscle mass, muscle atrophy, and functional loss. It represents a significant health problem in the elderly, since it is associated with slowing of movements, imbalance, pain, and serious injuries due to falls. There is currently no medical treatment available for sarcopenia. However, various management approaches have been proposed (reviewed in Rondanelli et al. (2016)) to delay sarcopenia progression including an adequate and balanced diet and regular exercise [133].

Myoblasts are the primary progenitor cells that differentiate in the embryonic phase and play an integral role in the development of muscle tissue. One study reported overproduction of ROS, an increase in oxidative stress markers, and a reduction in myogenic differentiation and proliferation capacity in senescent myoblasts [133]. In a more recent *in vitro* study, a standardized ginger extract containing 6-GN and 6-SG was shown to prevent cellular senescence and promote myoblast differentiation in myoblast cells [134]. Moreover, many studies demonstrated that ginger supplementation at a dose of 2-4 g daily may alleviate muscle pain related to vigorous exercise and reduce the loss of muscle strength [133]. These data suggest that ginger and its active nutraceuticals may have a beneficial role in sarcopenia by reversing the ageing of myoblasts, possibly through their antioxidant and anti-inflammatory activities, and may be effective for maintaining the overall skeletal muscle health.

### 3.10. Osteoarthritis

Osteoarthritis is the most common joint disease in the world, characterized by degeneration of cartilage, pain, inflammation, impaired mobility, and dysfunction, especially in older populations. Although osteoarthritis is not regarded as an inflammatory arthritis, its pathogenesis involves various degrees of inflammation in the cartilage and surrounding tissues. Old age constitutes a risk factor for osteoarthritis. Osteoarthritis is among the leading causes of disability and chronic pain especially in individuals over the age of 65 years [135]. Ginger extract was shown to inhibit the production of the inflammatory mediators PGE2 and NO in osteoarthritic chondrocyte cell cultures in one study and, also, to inhibit oxidative stress and apoptosis in IL-1*β*-induced C28I2 human chondrocytes [20, 136]. A very recent meta-analysis concluded that oral ginger supplementation is more effective in pain relief and improvement of joint function in patients with knee osteoarthritis as compared to placebo, possibly through its anti-inflammatory and antioxidant activities [137].

### 3.11. Skin Ageing

In addition to protecting the body from a large variety of external aggressors, the skin also has important cosmetic functions. Skin changes are among the most visible signs of ageing. Wrinkles, loss of elasticity, and laxity occur in the skin with ageing. Notably, long-term exposure to solar ultraviolet (UV) radiation is the major determinant for extrinsic skin ageing [138]. Fibroblast-derived elastase enzyme is known to contribute to the formation of wrinkles by causing loss of elasticity in the skin exposed to UV-B [139]. Ginger extract, previously shown to inhibit fibroblast-derived elastase, was reported to prevent UV-B-induced loss of skin elasticity when administered topically to mouse and rat skin [139]. In another study, a reduction in the signs of skin ageing was observed in individuals using a ginger oil body cream for four weeks, probably attributed to the antioxidant activity of the plant [140].

## 4. Side Effects of Ginger

Ginger acts directly on the gastrointestinal tract by increasing muscle tone and peristalsis via its anticholinergic and antiserotonin action. Ginger can ameliorate the central nervous system side effects associated with antiemetic drugs. Similar to onion and garlic, ginger extracts can inhibit blood coagulation *in vitro* [141–143]. Ginger has few adverse effects, and only mild side effects have been reported including heartburn and diarrhea. In large doses, ginger may increase antiprostaglandin activity and gastric exfoliation *in vitro* [142, 143].

## 5. Conclusion and Future Directions

Ageing is a complex process that is determined by multiple and interdependent genetic, cellular, and environmental factors. Ginger, one of the most commonly used natural products both for gastronomic and medicinal purposes, has documented antioxidant, anti-inflammatory, anti-infection, and chemopreventive properties. In this review, the effectiveness of ginger in the prophylaxis and treatment of several and diverse ageing-associated diseases, like gastrointestinal, cardiovascular, respiratory, and neurological diseases, as well as the underlying biological mechanisms, is discussed thoroughly. However, the number of studies on the effective dosage, pharmacodynamics, and pharmacokinetics of ginger, which can be used to delay ageing and prevent degenerative diseases, is rather limited. Therefore, additional studies on ginger as a powerful natural product need to be conducted so as to enhance our understanding of the role and mechanism(s) of ginger in the prevention of disease.

## Data Availability

The data used to support the findings of this study are all included and available within the article.
